# Arabidopsis dolichol kinase AtDOK1 is involved in flowering time control

**DOI:** 10.1093/jxb/erx095

**Published:** 2017-04-03

**Authors:** Yueh Cho, Chao-Yuan Yu, Yuki Nakamura, Kazue Kanehara

**Affiliations:** 1 Molecular and Biological Agricultural Sciences Program, Taiwan International Graduate Program, National Chung Hsing University and Academia Sinica, Taipei, Taiwan; 2 Institute of Plant and Microbial Biology, Academia Sinica, Taipei, Taiwan; 3 Graduate Institute of Biotechnology, National Chung Hsing University, Taichung, Taiwan; 4 Biotechnology Center, National Chung Hsing University, Taichung, Taiwan; 5 Muroran Institute of Technology, Muroran, Japan

**Keywords:** Arabidopsis, dolichol, dolichol kinase, flowering time control, endoplasmic reticulum, isoprenoids, membrane protein, protein glycosylation, shoot apical meristem

## Abstract

Dolichols are a class of isoprenoids that consist of highly polymerized and unsaturated long-chain isoprenes. They play crucial roles in protein glycosylation including *N*-glycosylation, because the oligosaccharide is assembled on a lipid carrier, dolichyl diphosphate. Arabidopsis DOLICHOL KINASE 1, At*DOK1* (At3g45040), encodes a functional dolichol kinase that is involved in plant reproductive processes. The expression of AtDOK1 is limited to highly pluripotent cells although protein glycosylation is thought to be required ubiquitously in the entire plant body. In this study, we further explored AtDOK1 functions by creating leaky knockdown mutants of *DOK1.* We used a microRNA-mediated gene suppression technique because knockout of *DOK1* causes lethality. The *DOK1* knockdown mutants showed an early flowering phenotype without any remarkable growth defect in vegetative tissues. Indeed, AtDOK1 was highly expressed in emerging shoot apical meristems as well as inflorescence and floral meristems. A subcellular localization study of DOK1 revealed that DOK1 was localized at the endoplasmic reticulum. Our findings suggest that the endoplasmic reticulum-localized catalytically active DOK1 is highly expressed in the meristems and is involved in the control of flowering time, possibly by post-transcriptional regulation including protein glycosylation.

## Teaser

The early flowering phenotype of *dok1* mutants and localization of DOK1 at meristems suggests the potential importance of dolichol kinase function in flowering time control.

## Introduction

Dolichols are a class of isoprenoids that consist of highly polymerized and unsaturated long-chain isoprenes (Swiezewska and Danikiewicz, 2005). They play crucial roles in protein glycosylation because dolichol phosphate mannose (Dol-P-Man) serves as a lipid carrier for the initial steps of *N*-glycosylation, *O*-mannosylation, and glycosylphosphatidylinositol (GPI) anchoring (Loibl and Strahl, 2013). Given that protein glycosylation is a pivotal mode of protein modification at the post-translational level, understanding dolichol function has importance beyond plant biology.

Among the different subclasses of isoprenoid, the function of dolichol and its biosynthesis have been poorly investigated. However, a number of recent reports have proposed that dolichol biosynthesis has a critical function in plant development. For example, *LEAF WILTING 1* (*LEW1*), a *cis*-prenyltransferase involved in dolichol biosynthesis, causes a lethal effect when knocked out (Zhang *et al.*, 2008). Mutations of *POLYPRENOL REDUCTASE2* (*PPRD2*), which converts polyprenol to dolichol, also showed a lethal effect due to male sterility (Jozwiak *et al.*, 2015). Furthermore, two lines of evidence have shown that the dolichol kinase *DOLICHOL KINASE 1*/*EVAN* (*DOK1*/*EVN*) has an essential function in plant reproductive processes (Kanehara *et al.*, 2015; Lindner *et al.*, 2015). While these evidences undoubtedly demonstrate that dolichols have an essential role in plant development, our previous characterization of the tissue-specific expression pattern of DOK1 found that DOK1 expression was limited to highly pluripotent cells (Kanehara *et al.*, 2015), although protein glycosylation is supposed to be required ubiquitously in the entire plant body. However, studying the function of DOK1 in such cell types is hampered by the fact that gene disruption of *DOK1* causes a lethal effect (Kanehara *et al.*, 2015; Lindner *et al.*, 2015).

In this report, we investigated DOK1 function by creating leaky knockdown mutants using a microRNA-mediated gene suppression technique (Schwab *et al.*, 2006). Leaky knockdown mutants of At*DOK1* showed early flowering phenotype without any remarkable growth defect in vegetative tissues. A subcellular localization study, in which the DOK1-Venus fluorescent reporter was stably expressed, revealed that DOK1 may be localized at the endoplasmic reticulum (ER), where protein glycosylation occurs. Indeed, AtDOK1 was highly expressed in the emerging shoot apical meristem (SAM) as well as in the inflorescence meristem (IM) and floral meristem (FM). However, no obvious changes were found in gene expression involved in flowering time control in the leaky mutants of At*DOK1*. Thus, we suggest that ER-localized catalytically active DOK1 is highly expressed in the SAM and is involved in the control of flowering time, possibly by post-transcriptional regulations including protein glycosylation.

## Materials and methods

### Plant material and growth conditions

*Arabidopsis thaliana* Columbia-0 (Col-0) ecotype was used in this study. Surface-sterilized seeds were planted directly onto Petri dishes containing half-strength Murashige and Skoog (MS) medium with 0.86% (w/v) sucrose. For normal growth conditions, plants were grown at 23°C under long-day conditions (LD; 16-h light /8-h dark cycle) with a light intensity of 150 μmol m^−2^ s^−1^ in a controlled culture room. Flowering time was compared between lines by the number of rosette leaves present at the time of bolting under LD conditions.

### Topology prediction

The membrane topology of AtDOK1 protein was predicted by the TMpred program (http://www.ch.embnet.org/software/TMPRED_form.html) using the amino acid sequence of AtDOK1 (UniProtKB: F4J4C8).

### Construction of plasmids and plant transgenic lines

The artificial microRNA (amiRNA) sequence targeting *DOK1* (At3g45040) was designed using WMD2 software according to the instructions available on the website (http://wmd2.weigelworld.org/cgi-bin/mirnatools.pl). The fragment for the hairpin structure containing miRNA (TGATGCTAATATTGGGCCCAG) and miRNA* (CTAGGCCCAATATAAGCATCT) was cloned by PCR using the plasmid vector pRS300 with the primers KK605, 606, 607, and 608 for the *amiDOK1-1* construct. The fragment for the hairpin structure containing miRNA (TCAATGACGTATTCACGCCAG)and miRNA* (CTAGCGTGAATACCTCATTGT) was cloned by PCR using the plasmid vector pRS300 with the primers KK609, 610, 611, and 612 for the *amiDOK1-2* construct. The amiRNA precursor fragments were then cloned into the *Xho*I and *BamH*I sites of pJL8, which contained 2 × *Pro35S* in the pENTR/D-TOPO plasmid (Invitrogen, Carlsbad, CA, USA) (Lin *et al.*, 2015) to obtain pCY33 and pCY35, respectively. The plasmids pCY33 and pCY35 were then recombined into the pBGW destination vector using LR Clonase (Karimi *et al.*, 2005) to obtain pCY39 and pCY36, respectively. The plasmids pCY39 and pCY36 were transformed into the wild-type plants by *Agrobacterium*-mediated transformation. Transformants were selected by 0.1% Basta solution on soil-growing seedlings. In total, 57 and 42 independent transgenic plants were examined for the *amiDOK1-1* and *amiDOK1-2* constructs, respectively. Lines 12 and 43 were selected for subsequent analyses of *Pro35S:amiDOK1-1* and lines 27 and 30 for *Pro35S:amiDOK1-2*. The point mutation (G471D) in AtDOK1 was created in pCC5 (pDO105-DOK1) by quick change site-directed mutagenesis (Sawano and Miyawaki, 2000) with the primer KK501 (Kanehara *et al.*, 2015). To insert the His-tag sequence in *SEC59* and *DOK1*, the quick change site-directed mutagenesis was performed with the primers YC173 and YC174, respectively. The template plasmids of non-tagged *SEC59* and *DOK1* for the quick change site-directed mutagenesis have been described previously (Kanehara *et al.*, 2015). The Venus fluorescent reporter construct of DOK1 was created by inserting a triple Venus cassette at the *Sfo*I site into pCC25 (*ProDOK1:DOK1* with *Sfo*I site) as described previously (Kanehara *et al.*, 2015). The obtained pCC33 (*ProDOK1:DOK1-Venus* in pENTR vector) was then recombined into a pKGW destination vector (Karimi *et al.*, 2005) using LR Clonase (Invitrogen). The resulting plasmid, pCC37, was introduced into *Agrobacterium* GV3101 to transform *dok1-1* heterozygous plants. Transformants were screened by kanamycin on a solid half-strength MS medium. In total, 57 independent transgenic plants were examined for Venus expression, and line 4 in the wild-type background was selected as the representative line for subsequent analyses. The primers used in this study are shown in Supplementary Table S1, available at *JXB* online.

### Yeast heterologous complementation assay

To assess functional complementation by observing the temperature-sensitive growth phenotype, *Saccharomyces cerevisiae sec59* mutant strain harbouring different plasmid vectors underwent serial dilutions on synthetic complete-Leu media, and were incubated for 2 days at the temperature indicated (Julius *et al.*, 1984; Heller *et al.*, 1992). The strain YCY1 was created to transform pDO105-AtDOK1[G471D] into the *sec59-1* mutant, KKY1111. The strains KKY1111, CCY5 (KKY1111 harbouring the empty vector), CCY6 (KKY1111 harbouring the pDO105-SEC59), and CCY7 (KKY1111 harbouring the pDO105-AtDOK1) have been described previously (Kanehara *et al.*, 2015).

### Quantitative reverse transcription polymerase chain reaction

Total RNA was isolated from seedlings at the time indicated using a plant RNeasy Mini Kit (Qiagen, Dusseldorf, Germany) according to the manufacturer’s instructions. RNase-free DNase (Qiagen) was used during the on-column digestion to remove genomic DNA contamination. The cDNA was synthesized by the SuperScript III First-Strand Synthesis System (Invitrogen) and used as the templates for quantitative reverse transcription (qRT) PCR analysis. *Actin2* (*ACT*) was used as the control. The sequences of oligonucleotide primers used in this study are listed in Supplementary Table S1. qRT-PCR was performed using the 7500 Real Time PCR System (Applied Biosystems, Foster City, CA, USA). The comparative threshold cycle method was used to determine the relative amount of gene expression, as described previously (Lin *et al.*, 2015). The mean and standard deviations were calculated from three biologically independent experiments with three technical replicates.

### Confocal laser-scanning microscopy

Venus expression in the *ProDOK1:DOK1-Venus* transgenic line at SAM, IM, and FM was observed under a microscope (LSM 510 Meta; Carl Zeiss, Jena, Germany) equipped with a C-Apochromat ×40 objective with 1.2 numerical aperture. For plasma membrane staining, seedlings were immersed in 10 μg/ml of FM4-64 (Invitrogen) for 10 min and observed with confocal microscopy. For ER staining, samples were immersed in 1 μM of ER-tracker Red (Invitrogen) for 10 min and observed with confocal microscopy. Images were captured using an LSM 510 v3.2 (Carl Zeiss) with filters for Venus (514 nm laser, band-pass 520–555 nm), FM 4-64 (543 nm laser, band-pass 560–615 nm), and ER-tracker Red (543 nm laser, band-pass 560–615 nm). LSM 510 META software was used to quantify the mean intensity of DOK1-Venus fluorescence at IM, stage 1 of FM, and stage 2 of FM (see Supplementary Fig. S1 at *JXB* online).

## Results and discussion

### AtDOK1 is localized at the endoplasmic reticulum

**Fig. 1.**
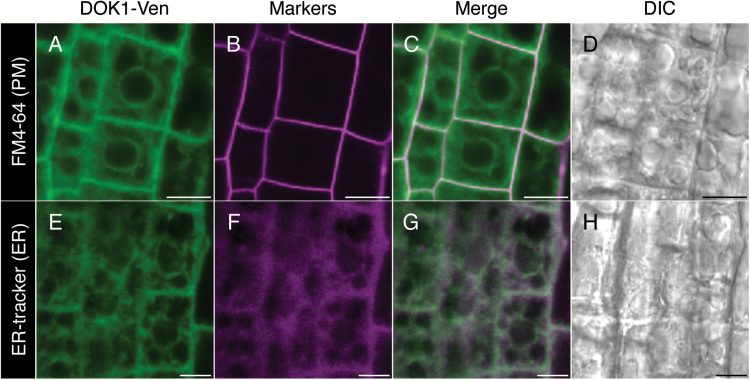
Subcellular localization of AtDOK1-Venus fluorescence in roots of 8-day-old *ProDOK1:DOK1-Venus* transgenic plants. (**A–D**) Fluorescence of *ProDOK1:DOK1-Venus* (A) and staining of the plasma membrane (PM) by FM4-64 dye (B) were merged in (C) with a differential interference contrast (DIC) image (D). (**E –H**) Fluorescence of *ProDOK1:DOK1-Venus* (E) and staining of the ER by ER-tracker Red (F) were merged in (G) with a DIC image (H). Scale bars, 10 μm.

To investigate the subcellular localization of AtDOK1 *in vivo*, we created transgenic Arabidopsis plants that stably expressed a DOK1-Venus fusion protein under the control of its own promoter (*ProDOK1:DOK1-Venus*). The triple repeat of the Venus fragment was fused C-terminally to At*DOK1*. We first transformed the *dok1-1/+* plants (whose T-DNA harbours a Basta-resistance marker) by *ProDOK1:DOK1-Venus* to obtain *ProDOK1:DOK1-Venus dok1-1/*-. After following up to the T_4_ generation, however, none of the lines showed 100% Basta resistance. As we reported previously, self-crossing of heterozygous *dok1-1* mutants produces <5% Basta resistance due to impaired penetration of the T-DNA (Kanehara *et al.*, 2015). In the T_4_ generation, we found a number of *ProDOK1:DOK1-Venus dok1-1* plant lines whose offspring showed ~60% Basta resistance, indicating a significant recovery of the population harbouring Basta resistance. These results suggested that the *ProDOK1:DOK1-Venus* construct partially complements the phenotype of *dok1-1*. We therefore used a wild-type background to stably express DOK1-Venus for subsequent analyses. To detect the subcellular localization of the DOK1-Venus fluorescent protein, roots of 8-day-old seedlings were observed for fluorescence, and the images were then merged with the staining pattern of either a plasma membrane marker, FM4-64 (Fig. 1A–D), or an ER marker, ER-tracker Red (Fig. 1E–H). As seen in Fig. 1, confocal laser-scanning microscopy observation revealed that Venus fluorescence co-localized with the ER-tracker, showing a typical ER-like shape that included the nuclear envelopes and a peripheral network near the plasma membrane. Venus fluorescence did not co-localize with the plasma membrane marker FM4-64. Although additional evidence, such as co-localization with known ER-localized fluorescent markers in Arabidopsis, may be needed for a clear conclusion, our observation was consistent with the subcellular localization of mammalian and yeast dolichol kinases (Heller *et al.*, 1992; Fernandez *et al.*, 2002). In addition, a transient assay using tobacco epidermis cells showed co-localization of DOK1 with ER marker proteins (Lindner *et al.*, 2015). These results suggest that AtDOK1 localizes at the ER membrane in Arabidopsis.

### Topology of AtDOK1 in the endoplasmic reticulum membrane

**Fig. 2.**
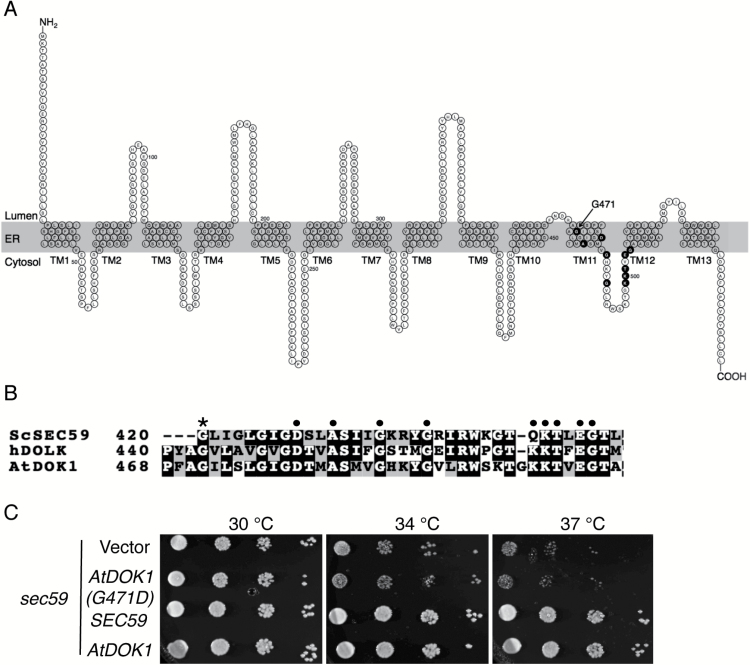
Topology of AtDOK1 in the ER membrane. (**A**) Predicted topological arrangement of AtDOK1 by the TMPred program. AtDOK1 contains 13 TM segments (TM1–TM13) with an ER luminally faced N terminus and a cytoplasmically faced C terminus. The DxxAxxxGxxxGx_8_KKTxEG motif conserved among the dolichol kinases of human, *S. cerevisiae*, and *Arabidopsis* are indicated by black closed circles with the alphabet written in white. (**B**) Amino acid alignment around the DxxAxxxGxxxGx_8_KKTxEG motif among *S. cerevisiae DOK* (ScSEC59), human DOK (hDOLK), and Arabidopsis DOK (AtDOK1). The conserved amino acid residues in the DxxAxxxGxxxGx_8_KKTxEG motif and Gly-471 residue in AtDOK1 are indicated with black dots and an asterisk, respectively. (**C**) Functional complementation assay of *S. cerevisiae sec59* mutant by *SEC59*, At*DOK1*, and At*DOK1 (G471D).* Cultures of *sec59* bearing *SEC59*, At*DOK1*, At*DOK1 (G471D*), or empty vector in SC-Leu media were serially diluted (10-fold dilution from left to right), and 5 μL of each was spotted on SC-Leu agar plates. The plates were incubated for 2 days under the temperature indicated.

The predicted AtDOK1 polypeptide comprises 569 amino acids (UniprotKB: F4J4C8), and is thought to be a multi-spanning membrane protein. Membrane topology analysis by the TMpred program predicts that AtDOK1 contains 13 transmembrane (TM) segments (TM1–TM13) with an N-terminus facing the ER lumen and a C-terminus facing the cytoplasm (Fig. 2A). The topological arrangement of AtDOK1 is similar to that of human dolichol kinase (hDOLK) (Shridas and Waechter, 2006). AtDOK1 contains a DxxAxxxGxxxGx_8_KKTxEG motif (black closed circles in Fig. 2B) that is conserved among enzymes utilizing CTP as a substrate, including hDOLK and yeast Sec59p (Fig. 2B) (Wu *et al.*, 1995; Shen *et al.*, 1996; Shridas and Waechter, 2006; Kanehara *et al.*, 2015). Based on the prediction, the motif DxxAxxxGxxxGx_8_KKTxEG will locate over a region in TM11 and the last cytoplasmic loop between TM11 and TM12 (black closed circles in Fig. 2A and black dots in Fig. 2B). This cytoplasmic loop has been thought to be part of a CTP-binding domain in cytoplasm (Shridas and Waechter, 2006). In addition to the DxxAxxxGxxxGx_8_KKTxEG sequence, the glycine residue G420 within TM11 in Sec59p (asterisk in Fig. 2B) is critical to DOK activity because substituting aspartic acid for G420 abolishes the DOK activity in a yeast *sec59-1* mutant (Heller *et al.*, 1992). To investigate whether a corresponding glycine residue is also important in AtDOK1, we mutated G471 to aspartic acid, which locates within TM11 (indicated in Fig. 2A), and transformed the *sec59* mutant with this AtDOK1 (G471D). The *sec59* mutant showed a temperature-sensitive growth phenotype (vector in Fig. 2C) (Heller *et al.*, 1992). The *sec59* mutant expressing either wild-type *SEC59* or At*DOK1* fully complemented the growth defect at 34°C and 37°C (Fig. 2C) (Kanehara *et al.*, 2015). However, the *sec59* mutant expressing At*DOK1* (G741D) failed to do so (Fig. 2C). We also performed the same complementation experiment by expressing His-tagged proteins in the *sec59* mutant. Although these proteins complemented the phenotype of the *sec59* mutant, we failed to detect the protein by western blotting, possibly because the proteins accumulated at low levels (Supplementary Fig. S1). Thus, these results indicate that G471 within TM11 in AtDOK1 is critical for DOK1 activity.

### Knockdown mutants of AtDOK1 showed an early flowering phenotype

**Fig. 3.**
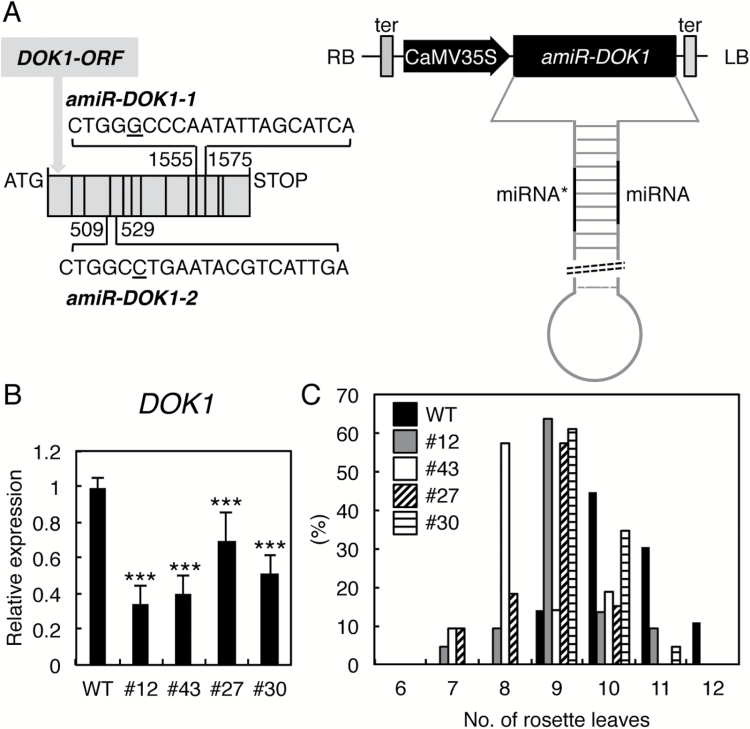
Characterization of the *Pro35S:amiDOK1* plants. (**A**) Schematic representation of the *Pro35S:amiDOK1-1* and *Pro35S:amiDOK1-2* constructions. Left: Grey boxes represent exons of At*DOK1* (At*DOK1*-ORF). The numbers 509/529 and 1555/1575 represent the position of the amiRNA target site by CTGGCCTGAATACGTCATTGA sequence and CTGGGCCCAATATTAGCATCA sequence for *amiDOK1-2* and *amiDOK1-1*, respectively. Right: The amiRNA precursor fragment designed to target At*DOK1* was cloned so that it is under the control of the cauliflower mosaic virus (CaMV) 35S promoter. LB, left border; RB, right border; ter, transcriptional terminator. (**B**) Relative expression level of At*DOK1* in the 7-day-old wild type (WT) and four independent transgenic seedlings harbouring *Pro35S:amiDOK1-1* (lines 12 and 43) and *Pro35S:amiDOK1-2* (lines 27 and 30) by qRT-PCR analysis. Data are shown from three biologically independent experiments with three technical replicates. Asterisks indicate statistical significance by Student’s *t*-test (***, *P* < 0.001). (**C**) The effect of knockdown of At*DOK1* on flowering time under LD conditions. The leaf number was counted when WT and *Pro35S:amiDOK1* plants (lines 12, 43, 27, 30) were bolting. Data are shown as percentages of total number of plants. Raw data are shown in Supplementary Table S2.

The *dok1* heterozygous mutants were defective in both male and female gametophyte developments and no homozygous mutants of *dok1* could be obtained (Kanehara *et al.*, 2015; Lindner *et al.*, 2015). To gain further insight into the role of AtDOK1 during plant development, we created knockdown mutants of At*DOK1* using an artificial microRNA-mediated gene suppression system expressed by the cauliflower mosaic virus 35S promoter (*Pro35S:amiDOK1-2*) (Schwab *et al.*, 2006). The artificial microRNA sequence of *amiDOK1-2* (CTGGCCTGAATACGTCATTGA) was designed to target a region at exon 3 and 4 of At*DOK1* (Fig. 3A). Among the 42 individual transgenic Arabidopsis plants we obtained, lines 27 and 30 were selected because of a moderate suppression of *DOK1* expression. In both lines, the expression level of *DOK1* was significantly reduced compared to that of the wild type on qRT-PCR analysis (Fig. 3B). We used mutant lines 27 and 30 for subsequent analysis.

When the knockdown mutants of *DOK1* were grown on soil, no obvious developmental defect was found in vegetative tissues. However, we noticed that both mutant lines 27 and 30 showed an early flowering phenotype compared to the wild type (Fig. 3). The number of rosette leaves was counted when plants were bolting under LD conditions. In the wild type, around 85% plants bolted when the leaf number was between 10 and 12, with an average of leaf number of 10.4 (Fig. 3C and Supplementary Table S2, available at *JXB* online). In contrast, the average leaf number was 8.8 for line 27 and 9.4 for line 30, with statistical significance (Supplementary Table S2). To exclude a possible off-target effect of the amiRNA sequence on this phenotype, we created another amiRNA-mediated gene suppression line (*Pro35S:amiDOK1-1*) (Fig. 3A). Whereas a number of transformants showed pleiotropic and sterile phenotypes, possibly due to an enhanced suppression of *DOK1*, we isolated two representative lines with moderate suppression of *DOK1* (lines 12 and 43). The expression levels of *DOK1* in these lines were about 40% of the wild type (Fig. 3B), and a significant early flowering phenotype was observed in both lines 12 and 43 (Fig. 3C and Supplementary Table S2), as was observed in the *Pro35S:amiDOK1-2* lines (27 and 30). We also compared the time to bolting in these transgenic lines (see Supplementary Fig. S2 at *JXB* online). Taken together, these results indicate that lower expression of *DOK1* leads to an early flowering phenotype in Arabidopsis.

### AtDOK1 in shoot apical meristem

**Fig. 4.**
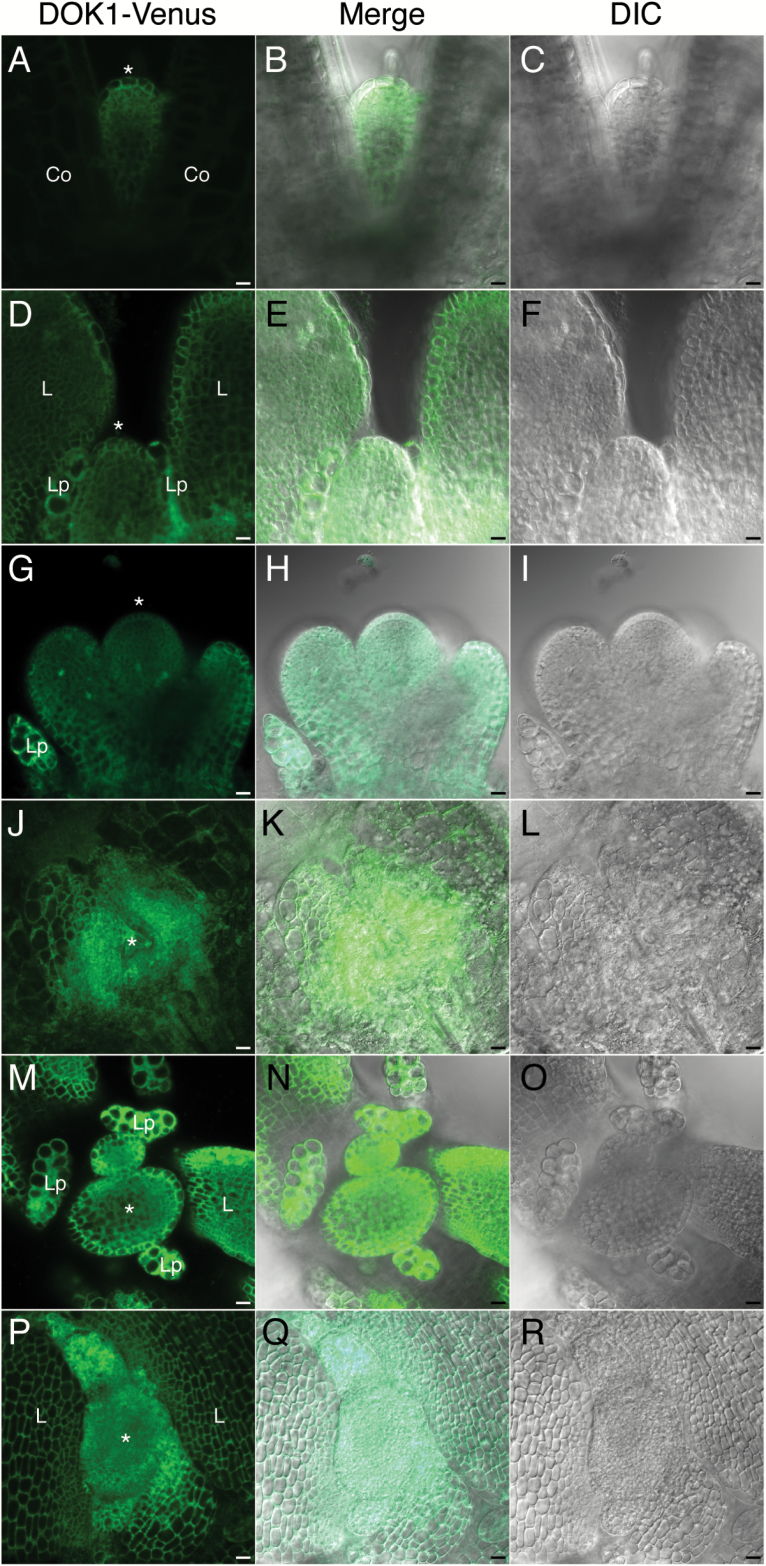
DOK1 expression at the SAM. (**A–C**) Side view of a 3-day-old *ProDOK1:DOK1-Venus* plant. Fluorescence of *ProDOK1:DOK1-Venus* (A) and differential interference contrast (DIC) image (C) were merged (B). (**D– F**) Side view of a 6-day-old *ProDOK1:DOK1-Venus* plant. Fluorescence of *ProDOK1:DOK1-Venus* (D) and DIC image (F) were merged (E). (**G–I**) Side view of a 12-day-old *ProDOK1:DOK1-Venus* plant. Fluorescence of *ProDOK1:DOK1-Venus* (G) and DIC image (I) were merged (H). (J-L) Top view of a 3-day-old *ProDOK1:DOK1-Venus* plant. Fluorescence of *ProDOK1:DOK1-Venus* (J) and DIC image (L) were merged (K). (**M–O**) Top view of a 9-day-old *ProDOK1:DOK1-Venus* plant. Fluorescence of *ProDOK1:DOK1-Venus* (M) and DIC image (O) were merged (N). (**P–R**) Top view of a 12-day-old *ProDOK1:DOK1-Venus* plant. Fluorescence of *ProDOK1:DOK1-Venus* (P) and DIC image (R) were merged (Q). Asterisks indicate the SAM. Co: cotyledon, Lp: leaf primordium, L: rosette leaf. Scale bars, 10 μm.

The early flowering phenotype we observed in the knockdown lines implies that At*DOK1* functions during the transition from the vegetative to reproductive phase. This prompted us to investigate AtDOK1 protein localization at the SAM using the DOK1-Venus plants, *ProDOK1:DOK1-Venus*. In 3-day-old seedlings, DOK1-Venus was expressed strongly in the SAM (asterisk in Fig. 4A and 4J). We further observed 6-day-old, 9-day-old, and 12 day-old seedlings. As can be seen in Fig. 4, DOK1-Venus was expressed in the SAM (asterisks in Fig. 4) in all stages observed. Notably, in addition to the SAM, strong fluorescence signals of DOK1-Venus were detected in the leaf primordia (Fig. 4D and M). These observations were consistent with our previous report in which GUS staining of *ProDOK1:DOK1-GUS* plants demonstrated that *DOK1* expression was limited to highly pluripotent cells (Kanehara *et al.*, 2015). These localizations suggest that AtDOK1 may play important roles in the SAM for the proper phase transition from vegetative to reproductive processes.

### AtDOK1 is expressed in inflorescence meristem and floral meristem

**Fig. 5.**
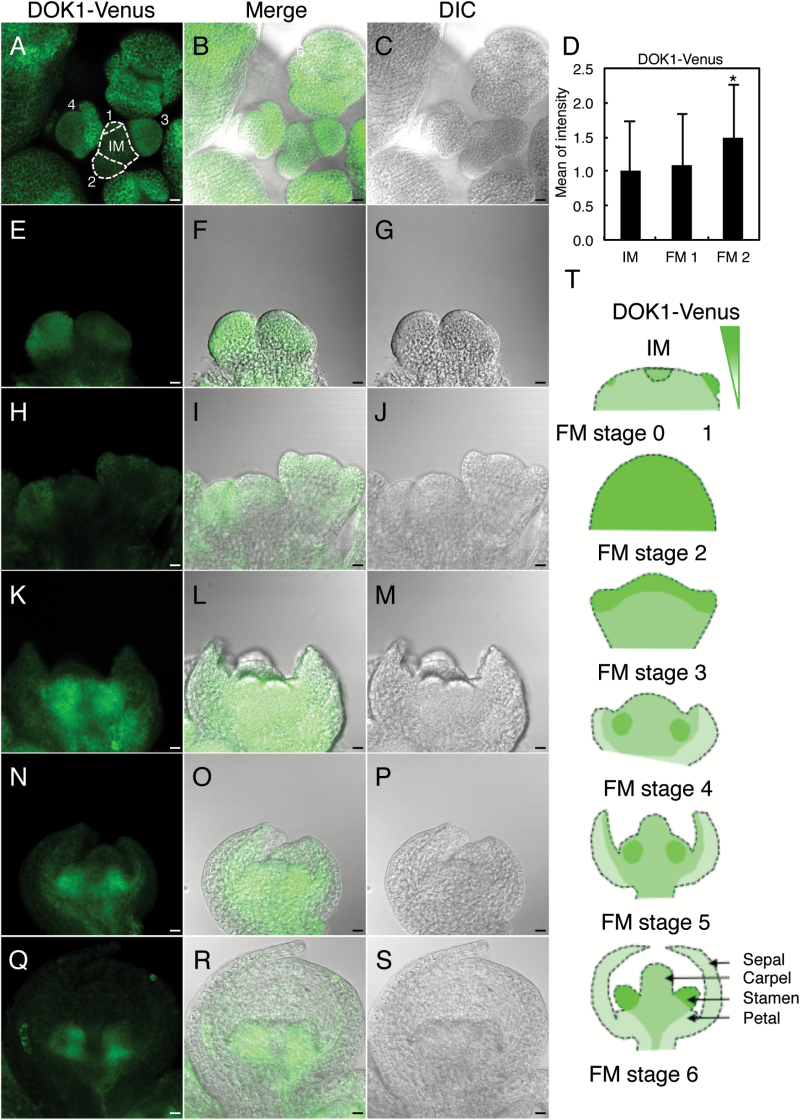
DOK1-Venus expression in the IM and FM in 5-week-old *ProDOK1:DOK1-Venus* plants. (**A–C**) Top view of a flower cluster in a *ProDOK1:DOK1-Venus* plant. Fluorescence of *ProDOK1:DOK1-Venus* (A) and DIC image (C) were merged (B). Buds of IM and stages of FM are indicated in A. (D) Quantification of DOK1-Venus fluorescence intensity in IM, and stage 1 and 2 of FM. DOK1-Venus intensity was higher in stage 2 of FM than in stage 1 or IM (Supplementary Fig. S1). The asterisk indicates statistical significance by Student’s *t*-test (*, *P* < 0.05). (**E–G**) Side view of IM and stage 2 of FM. Fluorescence of *ProDOK1:DOK1-Venus* (E) and DIC image (G) were merged (F). (**H–J**) Side view of stage 3 of FM. Fluorescence of *ProDOK1:DOK1-Venus* (H) and DIC image (J) were merged (I). (**K–M**) Side view of stage 4 of FM. Fluorescence of *ProDOK1:DOK1-Venus* (K) and DIC image (M) were merged (L). (**N–P**) Side view of stage 5 of FM. Fluorescence of *ProDOK1:DOK1-Venus* (N) and DIC image (P) were merged (O). (**Q–S**) Side view of stage 6 of FM. Fluorescence of *ProDOK1:DOK1-Venus* (Q) and DIC image (S) were merged (R). (**T**) Schematic representation of the expression patterns of *ProDOK1:DOK1-Venus* in IM and FM. Scale bars, 10 μm.

The *dok1* mutants were defective in pollen development, and a DOK1-GUS reporter assay previously demonstrated that DOK1 is expressed during early anther development (Kanehara *et al.*, 2015). To further investigate the roles of DOK1 during reproductive organ development in Arabidopsis, we examined DOK1 expression in 5-week-old *ProDOK1:DOK1-Venus* plants. Confocal microscopy revealed that DOK1-Venus fluorescence signals were strongly detected in the overall primordia of young flowers, including the IM and different stages of the FM, as can be seen in Fig. 5A–C. Quantification of fluorescence intensity of DOK1-Venus in the IM and stages 1 and 2 of FM showed that the DOK1-Venus fluorescence signal gradually increased during floral organ development (Fig. 5D and Supplementary Fig. S3, available at *JXB* online). DOK1-Venus was expressed in the whole IM, but the signal intensity was enhanced at stage 2 of FM compared with the intensity in the IM and stage 1 of FM (IM: the right-side bud in Fig. 5F, stage 2 of FM: the left-side bud in Fig. 5F). At stage 3 of FM, DOK1 was expressed more highly in the peripheral region (the right-side bud in Fig. 5I). However, as flower development progressed, the region showing fluorescence signals became limited to the centre of the FM from stage 4 to 6 (Fig. 5L, O and R). Ultimately, DOK1-Venus was expressed specifically in the stamen primordia (Fig. 5R). These observations were consistent with our previous results, in which DOK1-GUS was highly expressed in stage 8 anther and tapetum (Kanehara *et al.*, 2015). Thus, our results indicate that DOK1 is highly expressed in the IM and FM throughout flower development, particularly in the stamen primordia.

### Expression of flowering time control genes in amiDOK1 plants

**Fig. 6.**
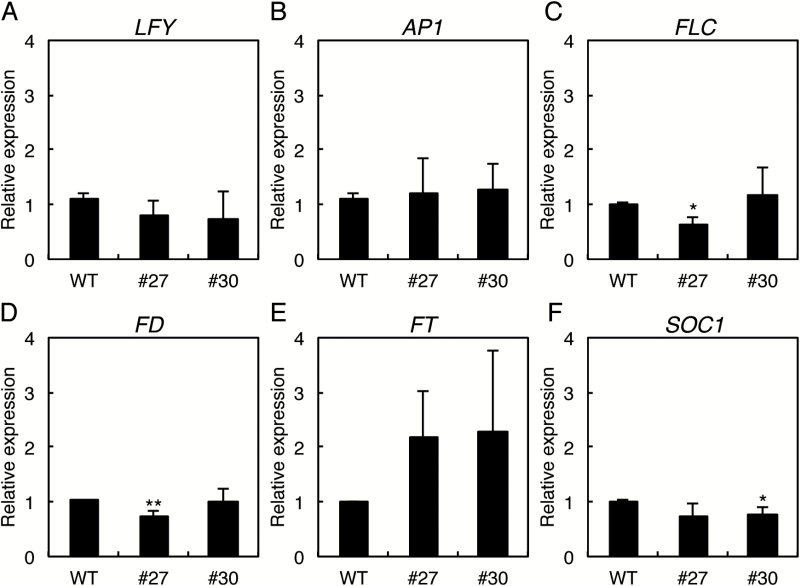
Expression of flowering time control genes in 14-day-old wild-type (WT) and *Pro35S:amiDOK1-2* transgenic plants (lines 27 and 30). Relative expression of (**A**) *LFY*, (**B**) *AP1*, (**C**) *FLC*, (**D**) *FD*, (**E**) *FT*, and (**F**) *SOC1* was analysed by qRT-PCR. Data are shown from three biologically independent experiments with three technical replicates. Asterisks indicate statistical significance by Student’s *t*-test (*, *P* < 0.05, **, *P* < 0.01).

Because of the early flowering phenotype found in the *DOK1* knockdown mutants and also the DOK1 expression in the SAM, we explored the possibility that DOK1 activity affects the regulation of flowering time. We first examined the expression of the flowering time control genes *LEAFY* (*LFY*), *APETALA1* (*AP1*), *FLOWERING LOCUS C* (*FLC*), *FD*, *FLOWERING LOCUS T* (*FT*), and *SUPPRESSOR OF OVEREXPRESSION OF CONSTANS1* (*SOC1*). *LFY* is an important factor in the transition from the vegetative to the reproductive phase (Blázquez *et al.*, 1997; Liu *et al.*, 2009), and directly activates *AP1*, which plays a role in specifying FMs and in determining the identity of perianth organs (Mandel *et al.*, 1992; Wagner *et al.*, 1999). In addition to *LFY*, *FLC* is also a key factor in the initiation of flowering, with the *flc* null mutant having an early flowering phenotype (Michaels and Amasino, 1999). The FLC protein binds FT and SOC1, both of which promote flowering in Arabidopsis (Helliwell *et al.*, 2006; Searle *et al.*, 2006). The FT protein is a component of florigen that transmits flowering signals from the leaf to the shoot apex. Moreover, a protein complex of FT and the transcription factor FD at the SAM activates downstream pathways for flowering (Abe *et al.*, 2005). In addition, recent studies revealed novel interactors for FT function, such as diurnally oscillating phospholipids and circadian-regulated *SODIUM POTASSIUM ROOT DEFECTIVE 1* (*NaKR1*) (Nakamura *et al.*, 2014; Zhu *et al.*, 2016). Here, we used 14-day-old seedlings of the wild type and the two *DOK1*-knockdown mutants, lines 27 and 30, to examine the expression of the above flowering time control genes. Although a slight change in gene expression was observed in some of the marker genes examined, no consistency was observed with regard to the gene expression profiles between the two knockdown mutants (Fig. 6). We also examined gene expression in 7-day-old seedlings; however, no significant change was observed in four independent *DOK1*-knockdown lines (see Supplementary Fig. S4 at *JXB* online). These results suggest that DOK1 may post-transcriptionally affect factors that regulate flowering time control because DOK is involved in protein glycosylation, including *N*-glycosylation, *O*-mannosylation, and GPI anchoring (Orlean, 1990; Loibl and Strahl, 2013; Kanehara *et al.*, 2015; Lindner *et al.*, 2015; Strasser, 2016). Indeed, several Arabidopsis genes involved in the *N*-glycosylation biosynthetic pathway (e.g. *TURAN* [*TUN*], *LEAF WILTING 3* [*LEW3*], *DEFECTIVE GLYCOSYLATION 1* [*DGL1*], and *STAUROSPORINE AND TEMPERATURE SENSITIVE 3-LIKE a/b* [*STT3a/b*]) are essential for plant viability, and mutations in these genes cause similar developmental defects to those we observed in the *DOK1* mutants (Koiwa *et al.*, 2003; Lerouxel *et al.*, 2005; Zhang *et al.*, 2009; Lindner *et al.*, 2015). In addition, mutations in *COMPLEX GLYCAN LESS 1* (*CGL1*) and *POLYPRENOL REDUCTASE 2* (*PPRD2*) resulted in defects in flowering time (Boyes *et al.*, 2001; Jozwiak *et al.*, 2015). A mutation in *GPI8*, which transfers an assembled GPI anchor to proteins, also changes the flowering time (Bundy *et al.*, 2016). Apart from the dolichol-linked intermediates in protein glycosylation, the Arabidopsis *SPINDLY* (*SPY*) that encodes an *O*-linked *N*-acetylglucosamine transferase is known to be a negative regulator of the gibberellin signalling pathway, which promotes flowering in Arabidopsis (Wilson *et al.*, 1992; Jacobsen and Olszewski, 1993; Silverstone *et al.*, 1997; Mouradov *et al.*, 2002). The *spy* mutants flowered earlier than the wild type and showed reduced fertility (Silverstone *et al.*, 2007).

In conclusion, we have shown that a dolichol kinase is involved in flowering time control in Arabidopsis. For future study, it will be interesting to investigate the role of DOK1 in other tissues/organs, such as roots, because of the characteristic tissue-specific expression pattern of DOK1 in Arabidopsis (Kanehara *et al.*, 2015).

## Supplemental data

Supplementary data are available at *JXB* online.

Fig. S1. Functional complementation assay of *S. cerevisiae sec59* mutant by *SEC59-6His*, At*DOK1-6His*, and At*DOK1(G471D)-6His.*

Fig. S2. Days to bolting in wild-type and *Pro35S:amiDOK1* plants.

Fig. S3. Quantification of DOK1-Venus fluorescence intensity at (**A**) IM, (**B**) stage 1 and (**C**) stage 2 of FM.

Fig. S4. Expression of flowering time control genes in 7-day-old wild-type, *Pro35S:amiDOK1-1*, and *Pro35S:amiDOK1-2* plants.

Table S1. Oligonucleotide primers used in this study.

Table S2. Flowering time of wild-type, *Pro35S:amiDOK1-1* and *Pro35S:amiDOK1-2* plants.

## Supplementary Material

supplementary_figures_S1_S4_tables_S1_S2

## Abbreviations:

amiRNA: artificial microRNA
DOK: dolichol kinase
ER: endoplasmic reticulum
FM: floral meristem
GPI: glycosylphosphatidylinositol
IM: inflorescence meristem
LD: long day
MS: Murashige and Skoog
SAM: shoot apical meristem
TM: transmembrane.
