# Supplementary material for: Arabidopsis dolichol kinase AtDOK1 is involved in flowering time control
Source: J Exp Bot. 2017 Apr 3;68(12):3243–52. doi: 10.1093/jxb/erx095 (PMC5853391; doi:10.1093/jxb/erx095)
Supplement: supplementary_figures_S1_S4_tables_S1_S2 [file erx095_suppl_supplementary_figures_s1_s4_tables_s1_s2.pdf]

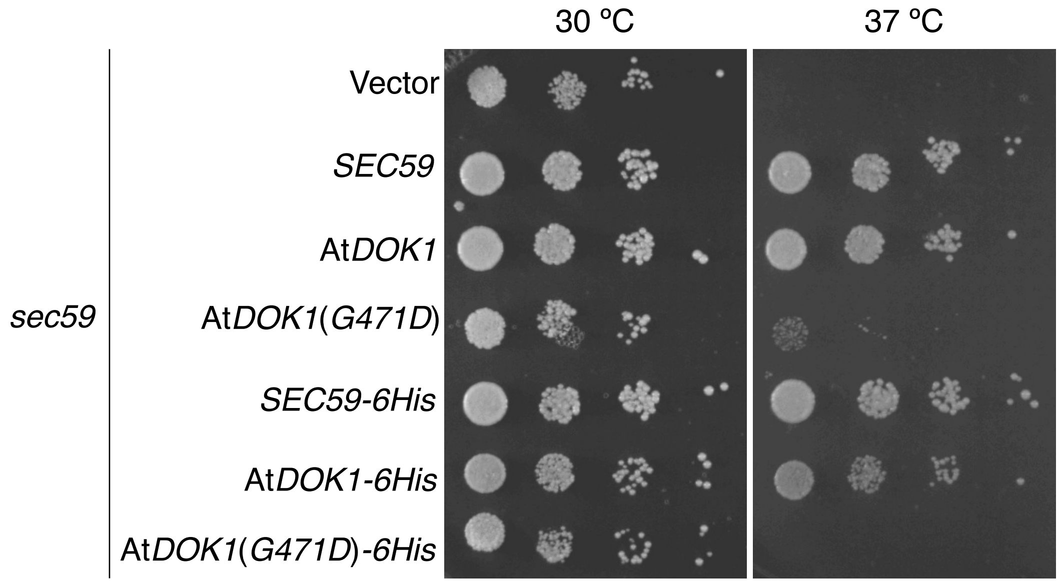

Fig. S1. Functional complementation assay of *S. cerevisiae sec59* mutant by *SEC59-6His,* At*DOK1-6His,* and At*DOK1*(*G471D*)*-6His.* Cultures of *sec59* bearing *SEC59*, At*DOK1*, At*DOK1*(*G471D*), *SEC59-6His*, At*DOK1-6His*, At*DOK1*(*G471D*)*-6His* or empty vector in SC-Leu media were serially diluted (10-fold dilution from left to right), and 5 µl each was spotted on SC-Leu agar plates. The plates were incubated for 2 days under the temperature indicated.

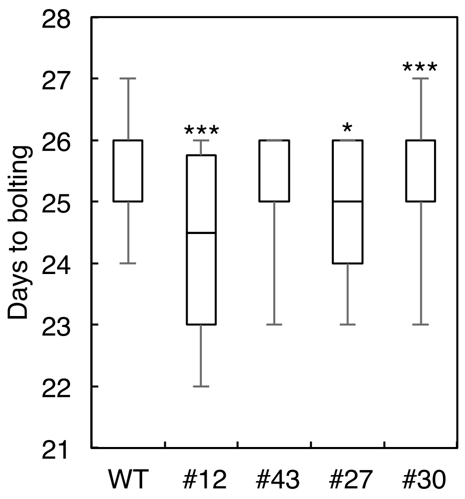

Fig. S2. Days to bolting in wild-type and *Pro35S:amiDOK1* plants. Days to bolting in the wild-type (WT) and *amiDOK1* (#12, 43, 27, 30) plants were shown as a boxplot with statistic significance (*, P<0.05; ***, P<0.001 by student t-test).

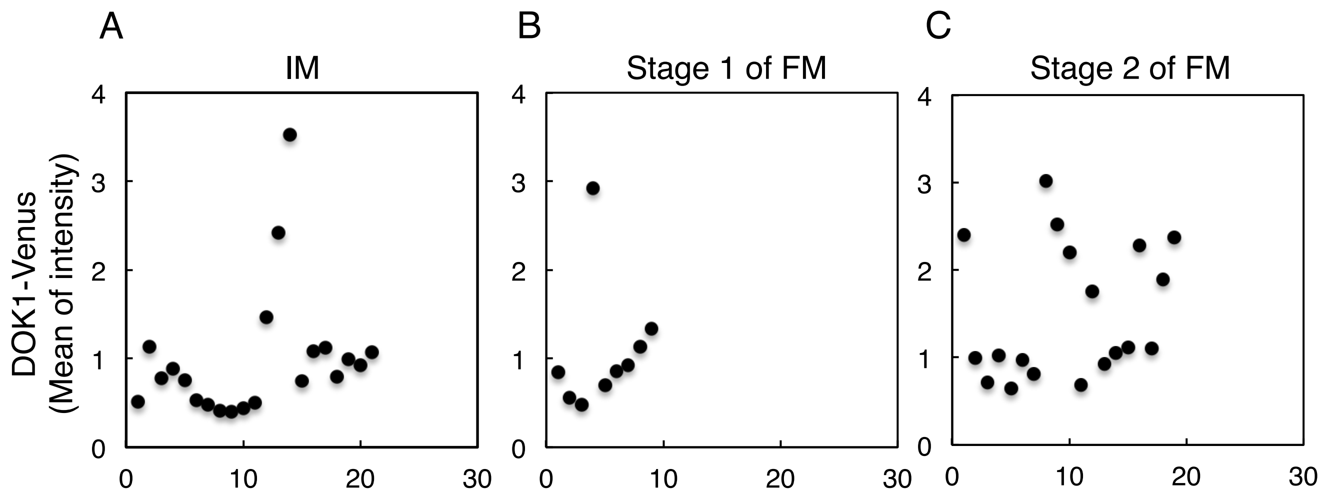

Fig. S3. Quantification of DOK1-Venus intensity at IM (A), stage 1 (B) and 2 (C) of FM. Five-week-old *ProDOK1:DOK1-Venus* plants were observed as shown in Fig. 5.

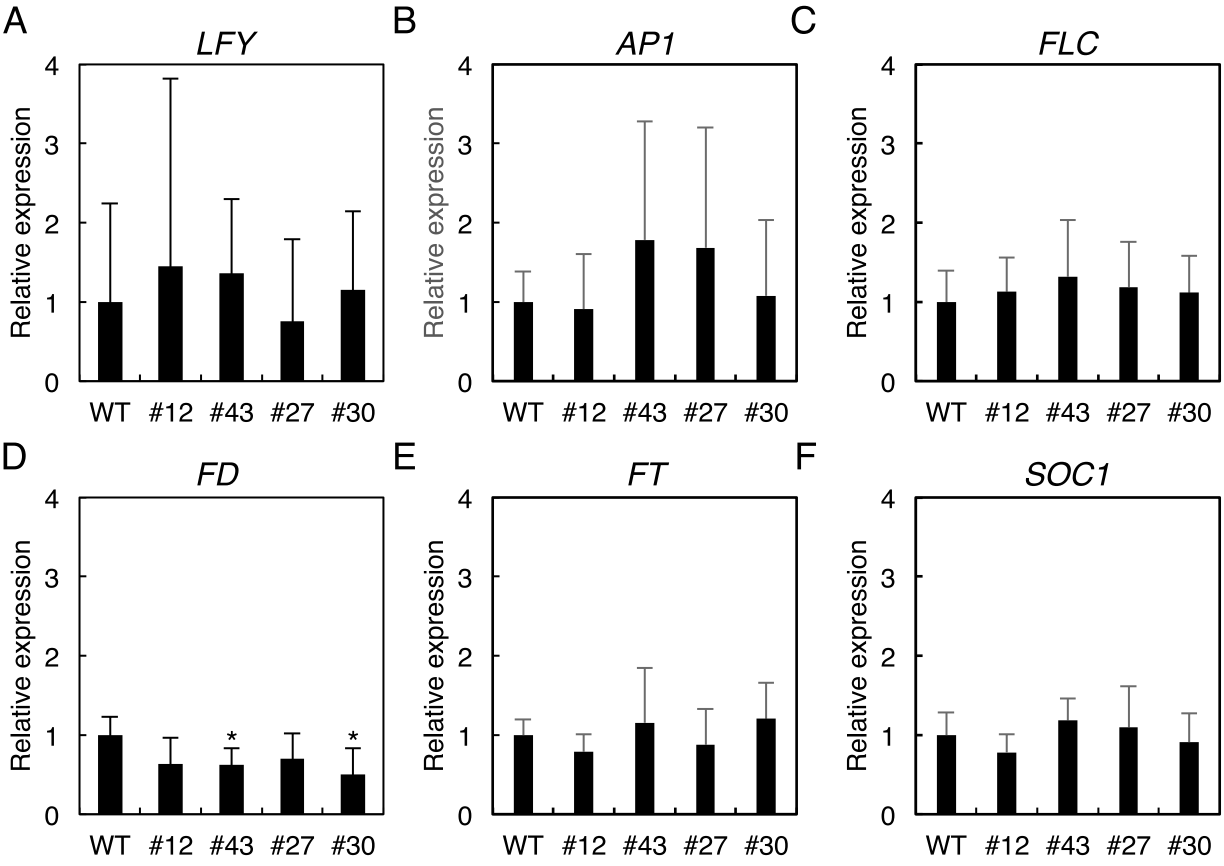

Fig. S4. Expression of flowering time control genes in 7-day-old wild-type, *Pro35S:amiDOK1-1* and *Pro35S:amiDOK1-2* plants. Relative expression of (A) *LFY,* (B) *AP1*, (C) *FLC,* (D) *FD,* (E) *FT*, and (F) *SOC1* was analyzed by qRT-PCR in 7-day-old wild-type (WT), *Pro35S:amiDOK1-1* (#12, #43) and *Pro35S:amiDOK1-2* (#27, #30) plants. Data were shown from three biologically independent experiments with three technical replicates. Asterisks indicate statistical significance by Student's *t*-test (*: $P<0.05$).

Supplemental Table 1. Oligonucleotide primers used in this study

Name	Purpose	Target gene	Sequences (5' to 3')
KK501	G471D point mutation	*DOK1*	GACCGAGCCTTATCTCCATTTGCTGACATTCTCAGCCTAGGAATTGGAGATAC
KK605	*amiDOK1-1*	*DOK1*	GATGATGCTAATATTGGGCCCAGTCTCTCTTTTGTATTCC
KK606	*amiDOK1-1*	*DOK1*	GACTGGGCCCAATATTAGCATCATCAAAGAGAATCAATGA
KK607	*amiDOK1-1*	*DOK1*	GACTAGGCCCAATATAAGCATCTTCACAGGTCGTGATATG
KK608	*amiDOK1-1*	*DOK1*	GAAGATGCTTATATTGGGCCTAGTCTACATATATATTCCT
KK609	*amiDOK1-2*	*DOK1*	GATCAATGACGTATTCACGCCAGTCTCTCTTTTGTATTCC
KK610	*amiDOK1-2*	*DOK1*	GACTGGCGTGAATACGTCATTGATCAAAGAGAATCAATGA
KK611	*amiDOK1-2*	*DOK1*	GACTAGCGTGAATACCTCATTGTTCACAGGTCGTGATATG
KK612	*amiDOK1-2*	*DOK1*	GAACAATGAGGTATTCACGCTAGTCTACATATATATTCCT
KK129	qRT-PCR	*ACT2*	GGTAACATTGTGCTCAGTGGTGG
KK130	qRT-PCR	*ACT2*	AACGACCTTAATCTTCATGCTGC
YC100	qRT-PCR	*DOK1*	AGGTTGAGAGAATCTTGCTTCG
YC101	qRT-PCR	*DOK1*	GAATGATCTCCAATGCAACAAA
YC102	qRT-PCR	*SOC1*	CTAAGGATCGAGTCAGCACCA
YC103	qRT-PCR	*SOC1*	ATTGAGCATGTTCCTATGCCTT
YC104	qRT-PCR	*LFY*	TCCTTCTTCAGGTCCAGACAAT
YC105	qRT-PCR	*LFY*	AACAGTGAACGTAGTGTCGCAT
YC106	qRT-PCR	*AP1*	ATGAGAGGTACTCTTACGCCGA
YC107	qRT-PCR	*AP1*	CAAGTCTTCCCCAAGATAATGC
YC108	qRT-PCR	*FT*	GCCAAAGAGAGGTGACTAATGG
YC109	qRT-PCR	*FT*	GGTTGCTAGGACTTGGAACATC
YC110	qRT-PCR	*FLC*	TAGAGCCAAGAAGACCGAACTC
YC111	qRT-PCR	*FLC*	ATCTCCATCTCAGCTTCTGCTC
YC112	qRT-PCR	*FD*	GCTGTGTTGTTGGTTCACTTCT
YC113	qRT-PCR	*FD*	AGAAGACGATGGTGATGAGGAT
YC173	Cloning for 6×His-tag	*SEC59*	TGTGAAAAATTAATTACTCTTCATCACCATCACCATCACTGACTGCAGGCATGCAAGCTT
YC174	Cloning for 6×His-tag	*DOK1*	TACTCACTCCTCTGCTTGCATCACCATCACCATCACTAAACGCGTCTGCAGGCATGCAAG

Table S1. Oligonucleotide primers used in this study

Supplemental Table 2. Flowering time of WT and transgenic plants under long-day condition

		Pro35S: amiDOK1-1		*Pro35S: amiDOK1-2*	
Genotypes	WT	#12	#43	#27	#30
#1	11	9	10	10	9
#2	10	11	9	9	9
#3	9	8	8	9	9
#4	11	8	8	9	10
#5	10	9	8	9	9
#6	9	9	8	8	9
#7	11	9	7	9	9
#8	12	9	8	7	9
#9	10	9	8	10	9
#10	12	9	8	8	9
#11	12	9	8	9	11
#12	11	11	9	9	10
#13	11	9	8	7	9
#14	11	10	8	8	9
#15	11	7	8	9	9
#16	11	9	10	9	9
#17	11	10	8	9	10
#18	12	10	7	10	9
#19	10	9	10	9	9
#20	9	9	10	9	10
#21	10	9	9	8	9
#22	11	9		9	9
#23	10			7	10
#24	10			9	9
#25	10			9	9
#26	10			9	10
#27	11			10	10
#28	10			10	10
#29	10			9	10
#30	10			8	9
#31	10			9	9
#32	10			9	10
#33	10			8	9
#34	9				11
#35	10				10
#36	9				9
#37					10
#38					9
#39					10
#40					9
#41					9
#42					10
#43					10
#44					10
#45					9
#46					9
Average	10.39	9.14	8.43	8.79	9.43
STDEV	0.87	0.89	0.93	0.82	0.58
t-*t*est (v.s. WT)		2.2E-06	8.4E-11	4.6E-11	7.4E-08

Table S2. Flowering time of wild-type (WT), *Pro35S:amiDOK1-1* (#12, #43) and *Pro35S:amiDOK1-2* (#27, #30) plants under long-day condition
